# Ge-Sb-Te Chalcogenide Thin Films Deposited by Nanosecond, Picosecond, and Femtosecond Laser Ablation

**DOI:** 10.3390/nano9050676

**Published:** 2019-05-01

**Authors:** Georgiana Bulai, Oana Pompilian, Silviu Gurlui, Petr Nemec, Virginie Nazabal, Nicanor Cimpoesu, Bertrand Chazallon, Cristian Focsa

**Affiliations:** 1 Integrated Centre for Environmental Science Studies in the North-East Development Region-CERNESIM, “Al. I. Cuza” University of Iasi, 700506 Iasi, Romania; georgiana.bulai@uaic.ro; 2 Université de Lille, CNRS, UMR 8523-PhLAM-Physique des Lasers, Atomes et Molécules, CERLA-Centre d’Etudes et de Recherches Lasers et Applications, Lille F-59000, France; oana.pompilian@inflpr.ro (O.P.); bertrand.chazallon@univ-lille.fr (B.C.); 3 National Institute for Lasers, Plasma and Radiation Physics, RO-077125 Magurele-Bucharest, Romania; 4 Faculty of Physics, “Al. I. Cuza” University of Iasi, 700506 Iasi, Romania; sgurlui@uaic.ro; 5 Faculty of Chemical Technology, University of Pardubice, 53210 Pardubice, Czech Republic; Petr.Nemec@upce.cz (P.N.); virginie.nazabal@univ-rennes1.fr (V.N.); 6 Université de Rennes 1, CNRS, ISCR (Institut des Sciences Chimiques de Rennes)–UMR 6226, F-35000 Rennes, France; 7 Faculty of Materials Science and Engineering, “Gheorghe Asachi” Technical University of Iasi, 700050 Iasi, Romania; nicanor.cimpoesu@tuiasi.ro

**Keywords:** pulsed laser deposition, chalcogenide thin films, Raman spectroscopy, spectroscopic ellipsometry

## Abstract

Ge-Sb-Te thin films were obtained by ns-, ps-, and fs-pulsed laser deposition (PLD) in various experimental conditions. The thickness of the samples was influenced by the Nd-YAG laser wavelength, fluence, target-to-substrate distance, and deposition time. The topography and chemical analysis results showed that the films deposited by ns-PLD revealed droplets on the surface together with a decreased Te concentration and Sb over-stoichiometry. Thin films with improved surface roughness and chemical compositions close to nominal values were deposited by ps- and fs-PLD. The X-ray diffraction and Raman spectroscopy results showed that the samples obtained with ns pulses were partially crystallized while the lower fluences used in ps- and fs-PLD led to amorphous depositions. The optical parameters of the ns-PLD samples were correlated to their structural properties.

## 1. Introduction

Important advances in nonvolatile solid state memory devices were driven by the discovery of Ge-Sb-Te (GST) alloys along the GeTe-Sb_2_Te_3_ tie-line in the mid-1980s [1]. Phase change (PC) memories are based on changes in optical properties and electrical conductivity of chalcogenide materials upon a rapid amorphous-to-crystalline phase transition and vice versa. These two states must present a high enough contrast in electrical resistivity or other (optical) parameters in order to be identified. The rapid changes from an amorphous (high electrical resistivity) to crystalline structure (low electrical resistivity) are induced by the Joule effect using an electric current pulse [2]. Depending on the intensity and duration of the pulses, the PC memory cells can be written or erased. The rapid laser-induced crystallization with large property changes represented the grounds for many research studies [3,4,5,6,7,8,9]. For applications in data storage devices, other properties such as a good thermal stability of the amorphous phase and the possibility of applying a large number of write-erase cycles need to be considered. The investigations of Yamada et al. [1] on 100-nm-thick GST films deposited by electron beam evaporation revealed that their crystallization temperatures were larger than room temperature but accessible for phase transitions by electric pulses. The laser-induced crystallization time of these samples was below 70 ns, which ensured a rapid recording. The degree of the optical change n,k (crystalline)-n,k (amorphous) on the GeTe-Sb_2_Te_3_ pseudo-binary line increases with an increasing Ge content [10], but GST chalcogenides with higher Sb concentrations present faster phase changes. Thus, the study of Ge-Sb-Te-based compounds in various compositions is essential when developing phase change devices with remarkable characteristics [11]. 

Several methods for chalcogenide thin film deposition have been employed to date such as spin coating [12], magnetron sputtering [13,14], thermal evaporation [15], atomic layer deposition [16], and metal organic vapor phase epitaxy [17]. Among these, the Pulsed Laser Deposition (PLD) technique is suitable for the thin film growth of complex materials with a good adhesion to the substrate and a high homogeneity. Chalcogenide thin films with a low surface roughness were reported in Reference [14]. The films deposited by PLD presented lower bandgap values than the samples obtained by sputtering [14]. Musgraves et al. [15] compared the structural, optical, and electrical properties of Ge-Sb-S thin films deposited by two methods: thermal evaporation (TE) and laser ablation. The chemical composition analysis revealed a slight variation of the Sb/S ratio from the stoichiometric value in the TE samples, while the PLD thin films replicated the atomic percentages of the main elements from the target. The refractive indices of the as-deposited (amorphous) PLD thin films presented higher values that the ones observed for the TE samples and even than the ones of the bulk material. PLD epitaxial Ge_2_Sb_2_Te_5_ thin films were obtained by Hilmi et al. [18]. However, their results also showed a decreased deposition rate as the substrate temperature was augmented, indicating a strong desorption during the deposition process. Similar observations were reported in Reference [19]. The studies done by Song et al. [20] and Boschker et al. [21] showed that the high adatom energy (proportional to the kinetic energy of the ejected particles that arrived at the substrate surface) during the pulsed laser deposition process influenced the stoichiometry and roughness of the film through preferential resputtering. However, photoexcited desorption [22] and in situ plasma plume diagnostics can offer information on the velocity of the ejected species [23,24,25,26].

This paper presents the main experimental results of an extensive systematic study on thin films of chalcogenide materials based on the ternary Ge-Sb-Te diagram. The films were deposited by laser ablation in various experimental conditions, varying laser parameters (pulse duration, repetition rate, wavelength, and fluence), target-to-substrate distance, and deposition time. The investigated materials were the endpoints of the GeTe-Sb_2_Te_3_ pseudo-binary line and the intermediate stable phases containing different proportions of these two structures: GeSb_2_Te_4_ (GST 124), GeSb_4_Te_7_ (GST 147), and Ge_2_Sb_2_Te_5_ (GST 225). 

## 2. Materials and Methods 

The Ge-Sb-Te thin films were synthesized by Pulsed Laser Deposition using an experimental setup described in detail in previous papers [24,27,28,29]. Two types of lasers were used for target ablation: a Nd-YAG laser (Continuum Surelite III-10) with a 10-ns pulse width and a 10-Hz repetition rate for which we used all four harmonics (266, 355, 532, and 1064 nm) and a Ti-Sa laser (Spectra Physics) with pulse durations of 2 ps and 120 fs and with a repetition rate of up to 1 kHz. The bulk materials (GeTe, GeSb_2_Te_4_, Ge_2_Sb_2_Te_5_, GeSb_4_Te_7_, and Sb_2_Te_3_) were prepared by the melt quenching method using high-purity elements (5N purity) and a melting temperature of 960 °C. The obtained polycrystalline targets were placed inside the stainless-steel vacuum chamber on a micrometric precision 3D-axis manipulator, while the substrate (single crystalline (100) Silicon and glass) was positioned at different distances in front of the target. The pressure during the depositions was kept in approximately the 10^−5^ Torr range using a turbomolecular pump. The other varied experimental parameters were the target-to-substrate distance (15–60 mm), fluence (0.1–10 J/cm^2^), and deposition time (5–60 min). Considering the numerous deposited samples, details on the growth conditions of each film are given as the paper proceeds. 

The morphological, compositional, structural, and optical properties of the synthesized films were studied using various techniques. The sample thickness was estimated using stylus profilometry (Dektak 6M). Images of the surface topography were obtained by optical microscopy (Olympus BXFM free-space confocal microscope, Olympus Europa, Hamburg, Germany) and scanning electron microscopy (Tescan Vega II LMH, Tescan, Brno, Czech Republic), using different magnifications. The chemical composition of the samples was studied by Energy-dispersive X-ray spectroscopy (EDS, Tescan Vega II LMH, Tescan, Brno, Czech Republic). Time of Flight-Secondary Ion Mass Spectroscopy (ToF-SIMS, ION-TOF 5, IONTOF, Münster, Germany) was used to analyze the distribution of the main elements on a 500 × 500 µm area on the sample surface in negative and positive polarity. In-depth profiles were obtained by sputtering 300 × 300 µm section with O_2_ (for positive polarity) or Cs (for negative polarity) ion beams and analyzing a 100 × 100 µm inner (centered) surface with ${Bi}_{3}^{+}$ ion beam (25 kV, 1 pA). Raman spectroscopy measurements were performed using an InVia Reflex spectrometer (Renishaw, 250 mm focal length, Renishaw SA, Champs-sur-Marne, France) equipped with an Ar^+^ laser source (514.5 nm wavelength, 36 mW laser power). Room temperature X-ray diffraction (Bruker AXS–Cu Kα radiation) patterns were required in the 5–65° 2*θ* range with 0.02° step and 5-s step times. The optical properties were investigated by variable angle spectroscopic ellipsometry (VASE, J.A. Woollam Co., Inc., Lincoln, NE, USA) in the 0.54–4.13 eV (2300–300 nm) spectral region.

## 3. Results and Discussion

### 3.1. Topography, Chemical Composition, and Structural Properties

#### 3.1.1. Nanosecond Laser Ablation

The optical microscopy images and thickness profiles revealed that the surface of the sample deposited by nanosecond laser ablation was affected by the presence of droplets, their density being dependent on the fluence. These microscopic particles deposited on the substrate/film surface can have several origins: dislodging of existing or laser-produced protruding target surface features, subsurface superheating, splashing of the molten surface layer, or condensation from vapor species due to supersaturation [30]. In femtosecond laser ablation, there are mainly nonthermal processes involved which end with the Coulomb explosion as the main ejection mechanism, while in nanosecond laser ablation, the longer pulse width leads to strong thermal effects. In this temporal range, the thermal mechanisms are predominant and determine the thermal damage of the lattice (homogeneous melting). 

Another important parameter that can have great influence on the microstructure of the deposited samples is the laser wavelength which, depending on the thermal properties of the material, can determine the ejection of different sized particles. Near-UV wavelengths offer higher photon energies and shorter penetration depths which can reduce the thermal effects when nanosecond pulse lasers are used. Thus, the deposition with the 266-nm laser radiation presents an advantage, especially when lower fluences are used. Large area depositions of GeTe were accomplished using the 266-nm radiation of the Nd-YAG laser and a lower fluence (1.2 J/cm^2^) compared to the other GeTe samples. The glass substrate was placed at a distance of 6 cm from the target, and the deposition time was 30 min. With these deposition conditions, improved results related to surface microstructure were obtained. The lower fluence used for ablation determined the deposition not only of a few droplets but also of a thinner thin film. However, one should consider that the thickness value of 120 nm was obtained when analyzing the ends of the deposited area but that the thin film can present a greater thickness in the center region due to the strong directionality of the ablation plume on the normal to the target surface. Although a larger target-to-substrate distance, a shorter deposition time, and a lower fluence were used to deposit the film on glass substrate, the thickness was still reasonable (120 nm) while the uniformity was improved. 

For a comparative study, Ge-Sb-Te thin films were deposited using the 266-nm laser wavelength in the same other conditions: laser fluence 3.81 J/cm^2^, deposition time 60 min, and target–substrate distance of 3 cm. Table 1 summarizes the stylus profilometer thin films thickness measurements and their elemental composition, as measured by EDS. The latter reveals a Ge over-stoichiometry in the GeTe samples and an increased Sb content in the Sb_2_Te_3_ and Ge-Sb-Te-based thin films. The concentration errors were approximately 1–2 at%. These main trends in composition variation were also observed when different conditions were used for thin film deposition. However, smaller deviations from the nominal values were found for the GeTe and Sb_2_Te_3_ samples deposited at higher target-to-substrate distances. For the intermediate compositions (GeSb_2_Te_4_, GeSb_4_Te_7_, and Ge_2_Sb_2_Te_5_), this content evolution with a target-to-substrate distance was not observed. 

A higher Ge concentration was also reported in References [6,31,32] for Ge-Sb-Te thin films deposited by PLD using a KrF excimer laser (248 nm, 30 ns, 20 Hz) at a fluence of 2.6 J/cm^2^. However, the recorded deviations were lower than the ones reported in this paper. The large deviations from the nominal composition in this study can be explained by the higher fluence used for target ablation. The high temperature induced at laser-target interaction could induce a more rapid evaporation of Te with respect to Ge, depending on the chemical properties of each species. The tellurium deficiency can be caused by its higher volatility compared to Ge or Sb [24].

**Figure 1:**
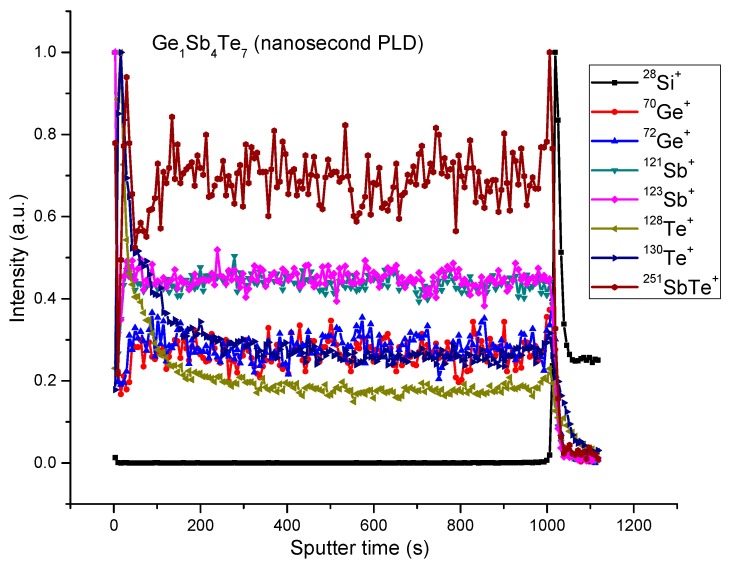
The ToF-SIMS depth profiles obtained in a positive polarity of the Ge_1_Sb_4_Te_7_ sample.

The distribution of Ge, Sb, and Te in the thin film volume was analyzed by ToF-SIMS depth profiling. Figure 1 presents the obtained profiles for the Ge_1_Sb_4_Te_7_ thin film. 

A uniform depth profile distribution was observed for Ge_1_Sb_4_Te_7_ thin films where three samples with different thicknesses were analyzed. However, an elevated Te content was observed at the region close to the thin film surface. This can come also from matrix effect due to oxidation at interface. As Te has a more pronounced metallic character, it can be more affected by this. The same behavior was observed in the GST 225 thin films deposited by Krusin-Elbaum et al. [33] by magnetron sputtering on Si substrate. Their work revealed that the deposited samples present a Te segregation on grain boundaries and surfaces. The composition of the main elements on the thin film surface was determined in Reference [32] by proton-induced X-ray emission (PIXE) and Rutherford back-scattering (RBS). 

**Figure 2:**
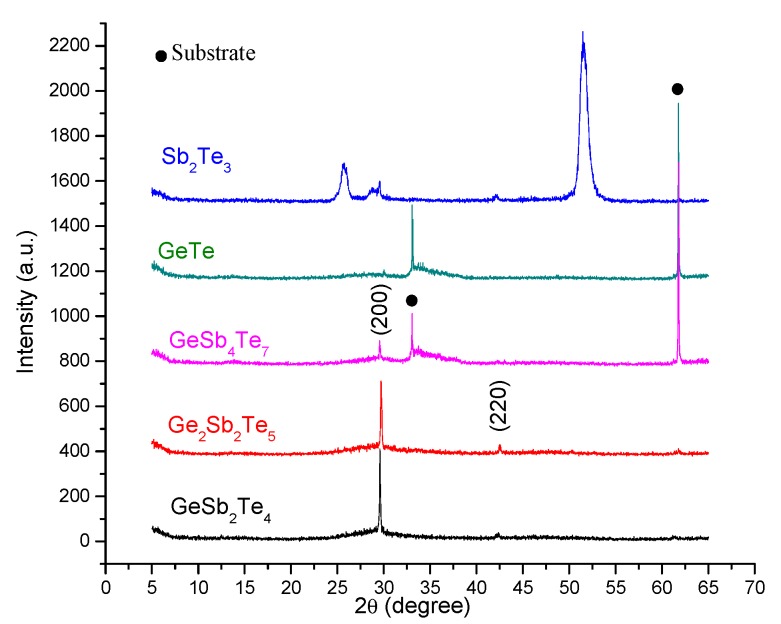
The XRD patterns of the chalcogenide thin films deposited using the 266-nm harmonic of the Nd-YAG laser.

Information on the structural properties of the deposited samples was obtained by Raman spectroscopy and X-ray diffraction. The XRD patterns of the GeSb_4_Te_7_, GeSb_2_Te_4_, and Ge_2_Sb_2_Te_5_ thin films indicated the formation of a face-centered cubic (fcc) crystalline structure (Figure 2). The peaks found at approximately 29° and 42° 2*θ* angles were correlated to the (200) and (220) diffraction lines of the cubic phase. The GeTe and Sb_2_Te_3_ samples presented a different behavior. While the XRD measurements for the GeTe film suggested an amorphous deposition, the ones for Sb_2_Te_3_ sample presented peaks that can be associated with two types of structures: one characterized by wider peaks (thus, smaller crystallite dimensions) and another one represented by the narrower diffraction line at the same 2θ angle as the fcc structure of the GST based samples. The first mentioned phase can be due to the excess of Sb in the Sb_2_Te_3_ sample. The larger diffraction lines from our XRD pattern were found at the same 2θ angles as the ones observed by Prokhorov et al. [34] when analyzing thin films of Sb-Te with a higher Sb atomic content. 

**Figure 3:**
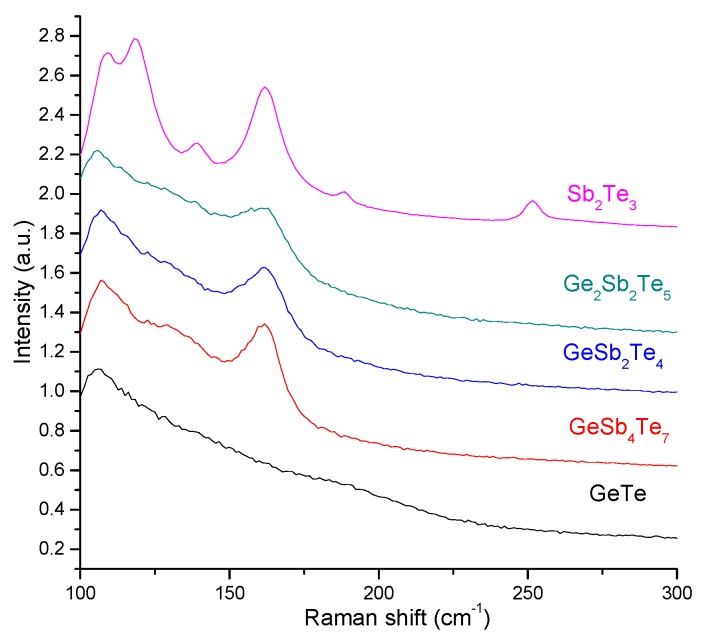
The Raman spectra of the five thin films deposited by ns-PLD at 266 nm (see Table 1 for deposition conditions).

The Raman spectra of the five thin films mentioned before (Table 1) are presented in Figure 3. The reported studies on Raman spectroscopy of GeTe materials revealed that the amorphous GeTe (a-GeTe) presents four peaks at 83, 125, 162, and 218 cm^−1^, and the crystalline GeTe (c-GeTe) shows dominant vibrational modes at about 80 and 120 cm^−1^. Moreover, Andrikopoulos et al. [35] observed several similarities between the peaks of the a- and c-GeTe samples. These were related to the much wider peak of the crystalline phase and to the narrower peak of the amorphous sample compared to the Raman response of other materials. These Raman features indicated that GeTe chalcogenide crystals present a distorted rock salt structure, while the a-GeTe seems more ordered than other glasses. In our study, the wide band in the 110–200 cm^−1^ region observed for the GeTe thin film is probably due to a combination between a crystalline structure and an amorphous phase. 

Sb_2_Te_3_ has a rhombohedral ($D_{3d}^{5}$ symmetry) structure, with the following centre of the Brillouin zone representation:

(1) $$\Gamma =2\left(A_{1g}+E_{g}\right)+3\left(E_{u}+A_{2u}\right)$$

where the ungerade(u)-modes are Raman active and gerade (g)-modes are IR active. Using density the functional perturbation theory, Sosso et al. [36] represented the IR and Raman spectra of crystalline Sb_2_Te_3_. The good agreement of their observations to the experimental vibrational spectra allowed them to assign each peak to specific phonons: E_g_ at 46 and 113 cm^−1^ and A_1g_ at 62 and 166 cm^−1^. A sketch of the displacement patterns of phonons is also presented in Reference [37]. Two peaks at 110 and 165 cm^−1^ were also observed by Nemec et al. [6] in the Raman spectroscopy study on Sb_2_Te_3_ bulk materials used as targets in the deposition process. In our case, the spectra recorded for this type of material are described by two peaks: 110 and 163 cm^−1^. In accordance with the data published in References [6,36,37], the first peak was attributed to the active Raman E_g_ mode, while the second one to the A_1g_ vibrational mode. However, for the Sb_2_Te_3_ film deposited using the 266 nm radiation of the Nd-YAG laser, additional vibrational modes were detected which were associated with the antimony-rich phase observed through XRD measurements. 

The Raman spectra of the Ge-Sb-Te based compounds indicated the formation of a crystalline structure, presenting two peaks at 110 and 160 cm^−1^. The same Raman response was obtained by Nemec et al. [6] when analyzing Ge-Sb-Te bulk materials. Based on the interpretation of the Raman spectra of GeTe and Sb_2_Te_3_ crystals, the bands of GST materials found at approximately 115–110 cm^−1^ and 165 cm^−1^ were assigned to the Γ_1_(A_1_), E_g_(2), and A_1g_(2) modes, respectively [6]. However, since the two peaks do not present a narrow width, we should consider the presence of an amorphous phase in the deposited samples, an observation that is sustained by the ellipsometry measurements as well. The presence of an amorphous phase can also be deduced from the XRD measurements where only the most intense peak of the fcc crystalline structure is observed.

#### 3.1.2. Femtosecond and Picosecond Laser Ablation

Several thin films of Ge-Sb-Te based materials were also deposited by fs- and ps-PLD using a Ti-Sa laser with a wavelength of 800 nm, 1.6 mJ laser energy, and 1 kHz repetition rate. Other experimental parameters were the deposition time (5 to 30 min), target–substrate distance (1.5 to 6 cm), and laser fluence (0.1 to 0.5 J/cm^2^). In the case of fs-PLD, the fluence used results in irradiance values in the approximate range of 1–4 TW/cm^2^. Although these values might seem quite high, comparable irradiances have been already used in other fs-PLD studies [29,38,39], leading to good quality thin films. The electron density was not measured in the current work; we note, however, that laser ablation of solid targets in similar pulse duration and irradiance conditions led to values well below the critical density (which is in the range of 10^21^ cm^−3^). For instance, Anoop et al. [40], using 40 fs pulses and fluences in the range 0.45–77 J/cm^2^, measured an electron density of the order of 10^17^ cm^−3^ close to the target. When the electron density was measured farther from the target (which can present more practical interest for PLD experiments), values in the range 10^10^–10^13^ cm^−3^ were observed [41,42,43].

**Figure 4:**
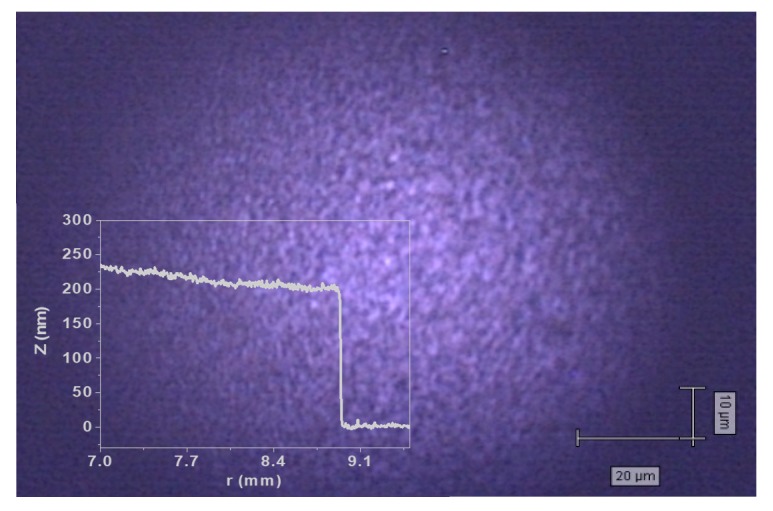
An optical microscopy image and surface topography of a Ge_2_Sb_2_Te_5_ thin film deposited by fs-PLD.

Compared to the thin films deposited by ns-PLD, the optical microscopy and stylus profilometry measurements revealed that these samples present more uniform surface, without large droplets (see Figure 4). In most cases, the thickness variation with the modified experimental parameters is evident. For example, a decrease of the GeTe sample thickness from 900 nm to 140 nm was observed when the laser fluence was decreased from 0.3 J/cm^2^ to 0.1 J/cm^2^. Also, a twofold (from 1400 nm to 700 nm) thinner GeTe film was obtained when the target–substrate distance was increased from 4 to 6 cm. 

Beside a more uniform surface, the Ge-Sb-Te based thin films presented also an improved chemical composition. Table 2 summarizes the representative Ge, Sb, and Te concentrations of three Ge_2_Sb_2_Te_5_ thin films deposited using lasers with different pulse duration. Deviations from the nominal (stoichiometric) concentrations were also recorded for the films deposited by fs- and ps-PLD; however, they were smaller (usually below 4 at%) than the ones observed for the ns-deposited thin films. 

**Figure 5:**
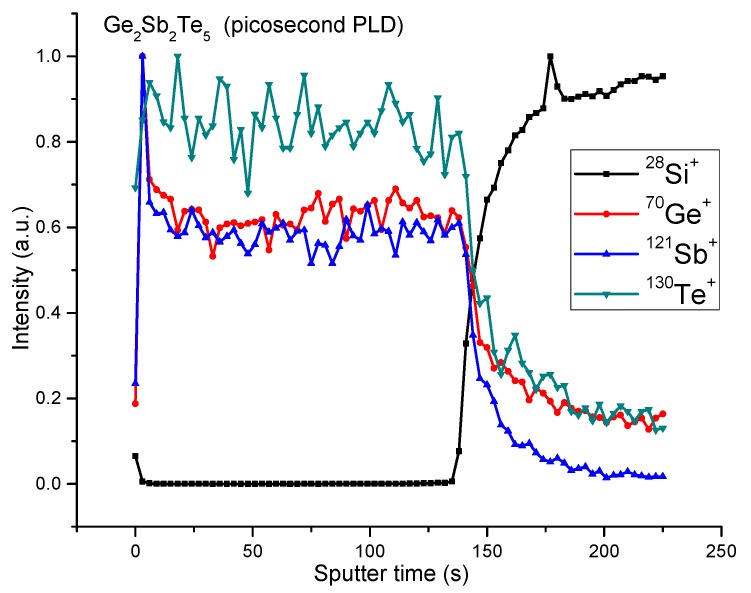
The ToF-SIMS depth profiles for the Ge_2_Sb_2_Te_5_ thin film deposited by ps-PLD (target-to-substrate distance = 3 cm, fluence = 0.3 J/cm^2^, deposition time = 60 min).

The elemental composition of the deposited samples was also probed by ToF-SIMS depth profiling. An example is given in Figure 5 for the Ge_2_Sb_2_Te_5_ thin film deposited by ps-PLD. As observed for the ns-PLD samples, a uniform distribution was recorded for the three elements. 

**Figure 6:**
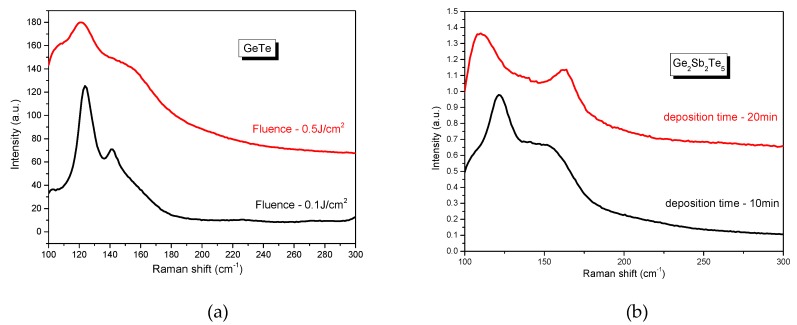
Raman spectra of the (**a**) GeTe and (**b**) Ge_2_Sb_2_Te_5_ thin films deposited in different conditions by fs-PLD.

The structural properties of the samples deposited using the Ti-Sa laser were analyzed using the same two methods mentioned in the previous section. The XRD patterns revealed an amorphous phase deposition for the Sb_2_Te_3_ and Ge_2_Sb_2_Te_5_ samples. Figure 6a presents the Raman spectra of two GeTe thin films deposited using different fluences of 0.1 J/cm^2^ and 0.5 J/cm^2^ (the target–substrate distance (6 cm), deposition time (30 min), and pressure (10^−5^ Torr) were kept constant). While the first film present two narrow peaks centered at 120 and 140 cm^−1^, the sample deposited using a higher fluence (thus, an increased thickness) showed a wider band with the maximum value positioned at 120 cm^−1^ which can be associated with the amorphous structure of the GeTe material [6]. For a clear assignment of the peaks found for the film deposited at 0.1 J/cm^2^, we took into consideration the Raman response of the Te phase. The bulk Te Raman spectra presents two peaks: one at 121 cm^−1^ which represents the A_1_ mode and a second one at 140.8 cm^−1^ which represent E_TO_ modes in crystalline Te-Te chain [44]. Considering that Te crystallizes at room temperature [45], a more adequate assignment of the two Raman peaks observed in our study would be based on Te segregation, which could be related as often reported to the photosensitivity of the GeTe film under laser irradiation of the Raman spectrometer when the film thickness is thinner rather than GeTe crystallization. We note, however, that parallel measurements using 785 nm excitation showed no difference in the resulting Raman spectra. Moreover, the main Raman feature of Ge is a vibrational mode at 300 cm^−1^. However, the peak from our study found at the same wavenumber is due to the contribution of the silicon substrate and not to Ge segregation, considering its high crystallization temperature (250 °C) [46].

Figure 6b shows the Raman spectroscopy results of two Ge_2_Sb_2_Te_5_ thin films deposited in 10 and 20 min respectively (the target–substrate distance (6 cm), fluence (0.2 J/cm^2^) and pressure (10^−5^ Torr) were kept constant). Again, a different Raman response was observed for the two samples with different thicknesses. While the first (10 min) film is characterized by an amorphous phase, the second one presents two peaks around 110 cm^−1^ and 160 cm^−1^ which could be associated with E_g_ and A_1g_ vibrational modes, respectively [6]. 

### 3.2. Optical Properties

Due to possible applications in phase-change optical storage and optical waveguides [47], an important parameter to be considered for this chalcogenide thin films is their reflectivity, which can be derived from [47,48]:

(2) $$R\left(E\right)=\frac{{\left(n\left(E\right)-1\right)}^{2}+k^{2}\left(E\right)}{{\left(n\left(E\right)+1\right)}^{2}+k^{2}\left(E\right)}$$

where *E*, *n*, and *k* are the photon energy, refractive index, and extinction coefficient, respectively (we recall that the refractive index is related to the complex dielectric constant for which the imaginary part can be measured experimentally and the real part can be evaluated using the Kramers–Krönig transformation). The optical properties (refractive indices and extinction coefficients) of several samples were investigated using variable angle spectroscopic ellipsometry (VASE, J.A. Woollam) with an automated rotating analyzer. 

**Figure 7:**
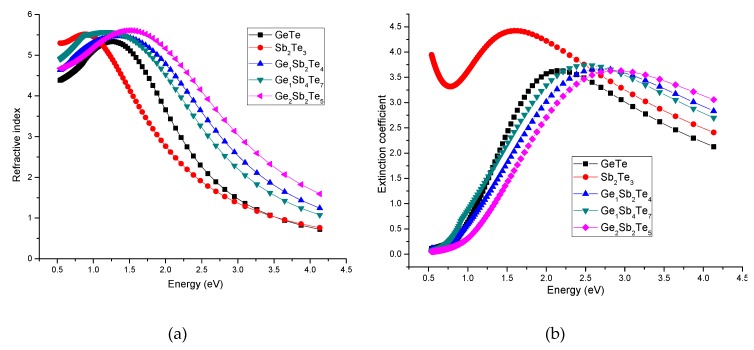

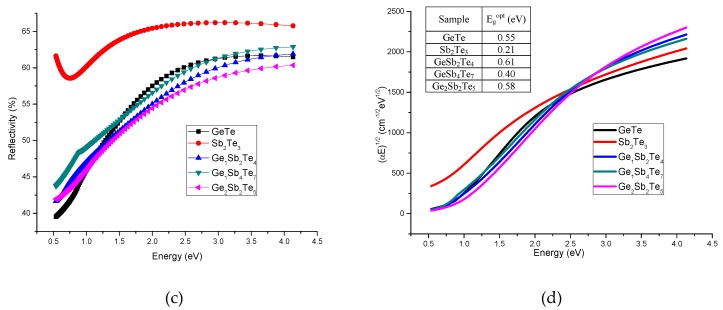
The ellipsometry results: the energy dependence of refractive index (**a**), extinction coefficient (**b**), reflectivity (**c**), and (αE)^1/2^ (**d**) for the Ge-Sb-Te based thin films deposited using the 266 nm radiation of the Nd-YAG laser.

When analyzing the Ge_2_Sb_2_Te_5_ series of thin films, we observed that the optical properties were not significantly influenced by the varied experimental parameters; thus, we continued by focusing on the Ge–Sb–Te thin films deposited in the same conditions. Figure 7a–c presents the spectral dependence of the refractive index, the extinction coefficient, and the reflectivity for the samples deposited by ns laser ablation. The optical band gap values were derived from the Tauc plots (αE)^1/2^ = f(E) (see Figure 7d). The absorption coefficient (α) was calculated using the well-known relationship with the imaginary part of the refraction index [49]:

(3) $$\alpha =\frac{4\pi k}{\lambda }$$

The obtained optical band gap values are presented in the inset of Figure 7d. 

Comparing these results with the ones reported by Nemec et al. [31,50], some similarities were observed: The refractive index presented an increase up to 1.2 eV and then a decrease for higher energies. The same type of dependence was observed for the extinction coefficient which reached its maximum value around 2.5 eV. However, the optical response of our samples presented features between those found for the amorphous and crystalline samples reported in Reference [31]. Comparing the plots of the refractive indexes, we observed higher maximum values than the amorphous chalcogenide thin films deposited by Nemec et al. [31] (and in the same time lower than the crystallized samples), but a more rapid decrease at large energy values was recorded, comparable with the optical features of the crystalline thin films. These results indicate a partial crystallization of our deposited samples. Moreover, we did not observe a clear dependence of the maximum values of the refractive index or extinction coefficient with the Sb_2_Te_3_ content. This can be explained by the different structural characteristics mentioned in the previous sections. Regarding the bandgap energies, our calculations (see inset Figure 7d) showed values comparable with the ones reported in Reference [31]. However, the poor microstructural quality of the deposited films led, in some cases, to slightly increased error values. Lee et al. [51] found that the optical bandgaps of their Ge_2_Sb_2_Te_5_ samples (deposited by RF magnetron) with amorphous, fcc, and hexagonal structures were 0.7, 0.5, and 0.5 eV, respectively, our data being closer to the values observed for the crystalline phase. An unusual value of the optical band gap was observed for the Sb_2_Te_3_ sample. However, the XRD results of this thin film revealed the presence of two phases, one of them induced by the increased Sb content. Preliminary ellipsometry measurements were also performed on fs and ps deposited samples, but due to the lower thickness of these samples, only the transparent region (up to 1 eV) could be investigated for the moment. These measurements will be extended in the near future using thicker fs and ps samples. Due to the narrow range in which we obtained a signal, no calculations of the E_g_^opt^ were allowed. 

## 4. Conclusions

Ge-Sb-Te thin films were deposited using various experimental conditions and lasers with ns, ps, and fs pulse duration. Analyzing the thin films deposited by nanosecond laser ablation, we observed that the sample thickness was influenced by the laser wavelength, fluence, target-to-substrate distance, and deposition time. Most of the deposited samples in this temporal regime presented droplets on the surface which decreased as the laser fluence was diminished. The EDS results revealed Te atomic percentages lower than the nominal value for all five compositions considered, which was explained by its lower vaporization heat. An over-stoichiometric Sb concentration was observed for the Ge-Sb-Te-based samples. The ToF-SIMS images and depth profiles revealed a uniform distribution of the main elements and of their combinations on the surface and in the volume of the films. The Raman spectroscopy and XRD analysis confirmed the formation of an fcc structure together with an amorphous growth for the Ge-Sb-Te based samples. While the XRD pattern of the GeTe thin film revealed an amorphous deposition, the Sb_2_Te_3_ thin film presented additional diffraction lines indicating multiphase formation. Ellipsometric measurements done on the Ge-Sb-Te thin films revealed *n* and *k* values between the ones of amorphous and crystalline samples reported in Reference [31], confirming the structural analysis results. The thin films deposited with femtosecond- and picosecond-pulsed lasers presented an improved morphology with no large droplets on the surface. Also, in their case, the thickness was found to be influenced by the deposition time, laser fluence, and target–substrate distance. The Ge, Sb, and Te concentrations obtained by EDS were much closer to the nominal values than the ones of the ns-PLD, their variations being smaller than the measurement error bar. The structural analysis results revealed an amorphous deposition for the Ge-Sb-Te-based fs-PLD films. The lower fluence used in fs-PLD determined the ejection of particles with lower kinetic energies than the ones generated by nanosecond ablation. This can influence the energetic transfer at substrate surface and thus the crystallization process. Moreover, on a fundamental background, the different ejection mechanisms involved for the various laser pulse durations (see above) favor a droplet formation in the case of ns-pulses vs a nanoparticle formation for ultrashort pulses. Overall, our results confirm that (high-repetition-rate) femtosecond PLD is a useful technique to obtain uniform, amorphous, and stoichiometric thin films in a short deposition time. 

## Figures and Tables

**Table 1:** The thickness (stylus profilometry) and elemental composition (EDS) of the thin films deposited using the 266-nm radiation with a 3.81 J/cm^2^ fluence, a 60-min deposition time, and a 3-cm target–substrate distance.

Target Nominal at% Composition	Thickness (nm)	EDS Measured Thin Film Composition (at%)		
		Ge	Sb	Te
GeTeGe _50_Te_50_	600	62.68	-	37.32
Sb_2_Te_3_ Sb_40_Te_60_	620	-	48.78	51.22
GeSb_2_Te_4_ Ge_14.28_Sb_28.57_Te_57.14_	600	13.14	35.78	51.08
GeSb_4_Te_7_ Ge_8.33_Sb_33.33_Te_58.33_	600	9.57	39.8	50.63
Ge_2_Sb_2_Te_5_ Ge_22.22_Sb_22.22_Te_55.55_	690	12.75	31.81	55.45

**Table 2:** Representative concentrations for Ge, Sb, and Te of three Ge_2_Sb_2_Te_5_ thin films deposited in different temporal regimes.

Pulse Duration	Deposition Conditions	Nominal Composition		
		Atomic %		
		Ge	Sb	Te
		22.22	22.22	55.55
Nanosecond	Nd-YAG laser (266 nm); Target-to-substrate distance = 3 cm; Fluence = 3.8 J/cm^2^ Deposition time = 30 min	25.23	26.81	47.97
Picosecond	Ti-Sa laser; Target-to-substrate distance = 4 cm; Fluence = 0.3 J/cm^2^; Deposition time = 60 min	23.45	21.69	54.86
Femtosecond	Ti-Sa laser; Target-to-substrate distance = 4 cm; Fluence = 0.3 J/cm^2^; Deposition time = 30 min	22.58	22.28	55.13
